# Recovery and Separation of Rare Earth Elements and Iron from Columbite Hydrometallurgical Effluent

**DOI:** 10.1021/acsomega.6c06153

**Published:** 2026-10-26

**Authors:** Gustavo Reis da Silva, Jorge Alberto Soares Tenório, Denise Crocce Romano Espinosa

**Affiliations:** Department of Chemical Engineering, University of São Paulo, Semi-Industrial Building, Block A, second Floor-250, Rua do Lago, Butantã, São Paulo 05508-080, Brazil

## Abstract

Rare earth elements exhibit unique physicochemical properties that justify their broad technological applications and rising global demand. Concerns about supply risks and resource scarcity have intensified the search for alternative supply routes and secondary sources. In this study, rare earth elements were recovered from an acidic effluent generated during the hydrometallurgical processing of columbite. Citric acid was used as a complexing agent to promote metal differentiation, and its effects on selective precipitation and solvent extraction were evaluated. Complexation with citric acid at a Fe:cit ratio of 1:1.5 and at pH 3 produced a precipitate with a rare earth-to-Fe ratio ten times higher than that of the initial solution. Subsequent solvent extraction with 10% v/v D2EHPA in kerosene recovered 92% of the remaining rare earth elements, yielding a separation factor of 27. The proposed process generated two streams enriched in rare earth elements.

## Graphical abstract

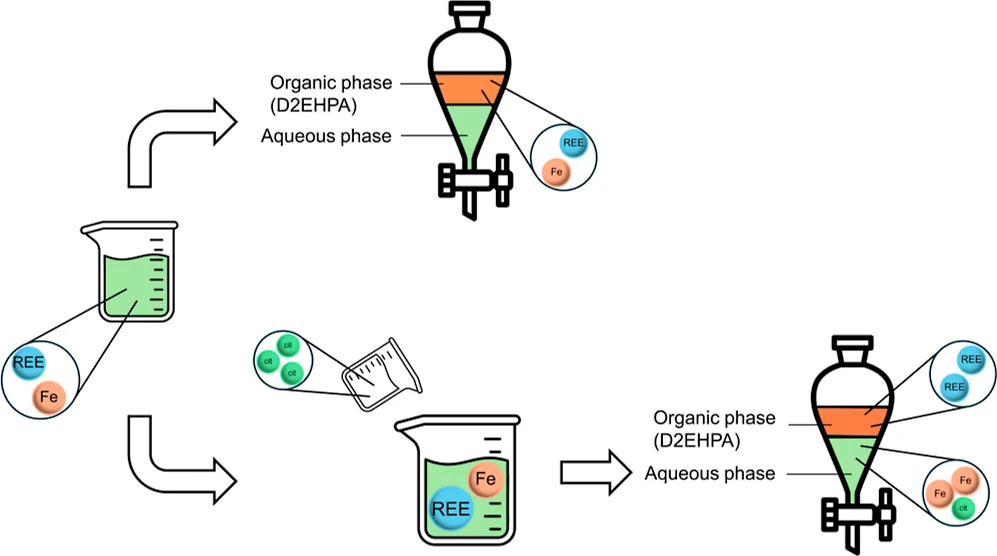

## Introduction

The group known as rare earth elements comprises the lanthanides together with yttrium (Y) and scandium (Sc). Despite their designation, these elements are more abundant in the Earth’s crust than elements such as silver (Ag) and mercury (Hg).^1^ These elements exhibit unique physicochemical characteristics and properties, which make them essential in the manufacture of a wide range of technologies, including catalysts, metallic alloys, and devices used in electronic equipment that has become indispensable in modern society.^2^

The main primary sources of rare earth elements are the minerals bastnäsite, monazite, and xenotime, although rare earth elements may also occur associated with other mineral phases. Bastnäsite may contain up to 70% rare earth oxides in its composition, predominantly cerium (Ce) and lanthanum (La), and rarely contains thorium (Th) in its structure. Monazite is a phosphate mineral commonly found in metamorphic rocks, but it can also occur in tin deposits; it is mainly composed of elements from the Ce group. Xenotime may also generally contain higher concentrations of heavy rare earth elements (HREE).^3,4^

Among secondary sources of rare earth elements, mining byproducts and wastes are particularly noteworthy, such as coal ash, red mud, and slags. In addition, waste electrical and electronic equipment (WEEE) that has reached the end of its service life represents an increasingly important source, as these materials can be subjected to recycling processes for rare earth recovery.^5–10^

The use of mining industry residues, such as leachate investigated in this study, represents a form of recycling and environmentally sustainable management, as it provides an alternative to disposal without recovery or treatment. By considering the process’ effluent as a secondary source of critical metals, value is effectively attributed to this material.

Processing rare earth element resources involves multiple hydrometallurgical operations, among which leaching is the initial and one of the most critical steps because it directly affects the efficiency of downstream purification and recovery processes. Mineral acids, such as H_2_SO_4_, HCl, and HNO_3_, are widely used for their high dissolution efficiency. However, growing environmental concerns have spurred research into organic acids as alternative lixiviants. Compared with conventional mineral acids, organic acids generally exhibit lower toxicity, higher biodegradability, lower corrosiveness, and higher selectivity for the targeted REE, making them attractive candidates for developing more sustainable hydrometallurgical processes.

Organic acids, including citric, oxalic, acetic, lactic, and tartaric acids, have recently attracted considerable attention as environmentally friendly lixiviants for recovering rare earth elements from secondary resources, such as red mud, coal fly ash, ion-adsorption clays, and NdFeB magnet scrap and residues.^11–16^ Several studies have demonstrated that these reagents can achieve high leaching efficiencies while offering additional advantages, such as reduced environmental impact and, in some cases, improved selectivity for rare earths. For instance, Ray et al. (2025)^11^ reported approximately 90% recovery of rare earth elements using a 2 M citric acid solution. Likewise, Petraglia et al. (2025)^17^ used acetic acid to leach end-of-life NdFeB permanent magnets and observed preferential dissolution of the rare earths, whereas most of the iron (Fe) remained in the solid residue. This selective dissolution reduced the Fe content in the pregnant leach solution, facilitating subsequent purification and separation.

The leaching performance of organic acids is primarily driven by the combined effects of proton-promoted dissolution and metal−ligand complexation. Initially, protonation weakens interactions between rare earth elements and the solid matrix, promoting their release into solution. Subsequently, the dissolved elements form soluble complexes with carboxylate ligands, shifting the dissolution equilibrium and facilitating metal recovery. Because this mechanism depends on mineralogical characteristics and the mode of occurrence of rare earth elements, organic acids generally exhibit higher leaching efficiencies for more reactive or weakly bound REE-bearing phases than for highly crystalline primary minerals.^18–20^

After leaching, the resulting pregnant leach solution typically contains both the target metals and a wide range of dissolved impurities, making downstream purification essential for producing high-purity products. Several separation techniques have been used for this purpose, including selective precipitation, solvent extraction, and adsorption.^21^ Selective precipitation relies on reagent addition and pH adjustment to form insoluble compounds; however, its efficiency may be compromised by coprecipitation of impurities.^22–24^ Solvent extraction is one of the most widely used purification techniques and relies on the selective transfer of metal species from the aqueous phase to an immiscible organic phase containing a suitable extractant. Adsorption using ion-exchange resins has also been extensively investigated for its high selectivity, particularly in dilute solutions.^25–28^ Nevertheless, achieving high selectivity remains challenging when chemically similar elements or high concentrations of impurities coexist in solution. In such cases, the addition of complexing agents has emerged as an effective auxiliary strategy to selectively mask interfering metal ions, modify metal speciation, and improve separation efficiency during downstream purification.^29–32^

Although organic acids offer environmental advantages over conventional mineral acids, their potential industrial applications must also be evaluated from economic and process-integration perspectives. Factors such as reagent cost, availability, consumption, recovery and reuse, and treatment of resulting aqueous streams can significantly affect the feasibility of large-scale implementation. Organic acids are viable in hybrid or selective flowsheets, especially where environmental performance and separation quality are critical. Considering the utilization of organic acids as an auxiliary complexing agent in downstream purification processes, its application may require lower reagent consumption compared to processes where organic acids serve as the primary leaching agents.^33,34^

The ongoing advancement of more efficient recovery methods and innovative separation techniques is especially crucial because rare earth elements are deemed critical raw materials by the European Union, given their significant economic value and the risks associated with their supply.^35,36^ These elements are indispensable across numerous industrial sectors, including renewable energy technologies, electric vehicles, advanced electronics, and defense applications, yet suitable substitutes are often unavailable or perform inferiorly. Therefore, developing sustainable recovery routes from secondary resources, alongside the exploitation of primary mineral deposits, is essential to diversify the supply chain, reduce resource dependence, and meet the growing global demand for rare earth elements.^37^

Thus, developing efficient and sustainable recovery routes for rare earth elements is not only essential to diversify the supply of critical raw materials but also contributes to global sustainability initiatives.^38–40^ In this regard, the recovery of rare earth elements from such sources is aligned with the United Nations (UN) 2030 Agenda for Sustainable Development, particularly Sustainable Development Goals (SDGs) 9 and 12.^38,40^ By promoting the valorization of industrial residues through innovative hydrometallurgical approaches, rare earth elements recovery contributes to SDG 9 (Industry, Innovation and Infrastructure), especially Targets 9.5 and 9.b, which encourage scientific research and technological innovation. In addition, the recovery of valuable metals from industrial wastes supports SDG 12 (Responsible Consumption and Production), particularly Targets 12.4 and 12.5, by fostering environmentally sound waste management, recycling, and resource efficiency.

The hydrometallurgical processing of columbite generates a sulfate leach liquor containing substantial concentrations of both rare earth elements and impurities, making the subsequent purification stage crucial for efficient metal recovery. Because Fe exhibits chemical behavior similar to that of rare earth elements under certain extraction conditions, its coextraction can significantly reduce process selectivity and compromise the purity of the recovered products.

Despite the widespread application of solvent extraction for rare earth element purification, the selective separation of these elements from authentic hydrometallurgical liquors remains a significant challenge due to the high concentrations of dissolved impurities commonly present in these solutions, particularly Fe.

In this context, the use of organic acids as auxiliary complexing agents during downstream purification represents a promising strategy to improve solvent extraction performance. By selectively modifying the aqueous speciation of impurity elements, these reagents can suppress their extraction while preserving favorable conditions for rare earth recovery. Accordingly, this study investigates the application of citric acid as a masking agent to selectively complex Fe in a real sulfate leach liquor obtained from the hydrometallurgical processing of columbite prior to solvent extraction with D2EHPA. The proposed approach aims to improve the separation between Fe and rare earth elements, contributing to the development of more selective and sustainable hydrometallurgical processes for the recovery of critical raw materials from secondary resources.

## Methodology

### Columbite Leachate Characterization

The columbite leachate studied in this study was provided by a Brazilian mining company. The original columbite concentrate underwent industrial processing involving two consecutive sulfuric acid leaching stages, followed by multiple water-washing steps. This processing route was designed to remove impurities while concentrating Nb and Ta for future alloy production.

The elemental chemical analysis of the leach liquor was performed using inductively coupled plasma optical emission spectroscopy (ICP-OES, Agilent Technologies 700) and energy-dispersive X-ray fluorescence spectrometry (EDXRF, Shimadzu 7200). Before analysis, the liquor was vacuum filtered through a membrane filter (47 mm diameter, 0.45 μm pore size) to remove any suspended solids that could interfere with the chemical analysis and damage the instruments.

During leach liquor characterization, pH was measured with a pH meter, and oxidation−reduction potential (ORP) was assessed using an ORP meter.

### Simulation of the Solution

FactSage 8.1 software was used to support understanding of the sample composition through thermodynamic equilibrium simulations using the Equilib module and the FACT PS database.

Simulations were performed using the Hydra/Medusa software packages. Hydra includes a thermodynamic database with log *K* values at 25 °C and was used to define the chemical system of interest. Medusa was used to generate various types of diagrams.

### Oxidation−Reduction Approach

To evaluate selective precipitation of rare earth elements without Fe coprecipitation, the oxidation−reduction (redox) potential of the liquor was reduced.

The initial approach involved keeping all Fe as Fe^2+^ through the addition of a reducing agent, followed by increasing the pH to facilitate the precipitation of rare earth elements.

Sodium dithionite (Na_2_S_2_O_4_) was used to adjust the solution’s redox potential. Its standard potential is −0.66 V. The reagent was gradually added to the leach liquor under stirring until the desired potential was reached. Afterward, the liquor was filtered to remove any precipitates or suspended solids, preventing them from moving to the selective precipitation step. The precipitation process was conducted selectively by adding NaOH microbeads as the precipitant until the solution pH reached 1, 2, and 4.5 at room temperature.

### Addition of a Complexing Agent and pH Adjustment

Following the experiments involving redox potential reduction and pH adjustment to promote the selective precipitation of rare earth elements, an alternative method was explored to separate lanthanides from Fe. This approach involved incorporating a complexing agent that functions as a masking agent within the solution, with citric acid chosen specifically as the ligand.

The first experiment involved adding citric acid in a 50% excess over the Fe concentration in solution at room temperature, while respecting solubility limits, because other components of the sample could also form complexes.

The reagent was slowly added to the solution under continuous stirring until the solid was completely dissolved. After the addition was complete, the solution was stirred for approximately 30 min to ensure that the reactions proceeded to completion and that none of the added reagents remained in the solid form.

The next step in the complexation study involved adjusting the solution pH to 1, 2, and 3 using NaOH to determine if the complexes formed solely with Fe. These pH levels were chosen based on literature, as they cover the precipitation range of rare earth elements in sulfate media and the pH at which Fe complexes are formed with the reagent used.^41,42^

After the addition of the complexing agents and the precipitating reagent, the solutions were filtered, stored, and diluted for chemical analysis by ICP-OES, and subsequently subjected to solvent extraction experiments.

The second group of experiments evaluated the behavior of the complexing agent, citric acid, under stoichiometric and substoichiometric Fe conditions (Fe:cit = 1:1 and Fe:cit = 1:0.8, respectively).

### Solvent Extraction Experiments

After the complexation experiments, the liquors were subjected to solvent extraction. For this purpose, D2EHPA, Cyanex 923, and Aliquat 336 were selected for their distinct extraction mechanisms, namely cationic, solvation, and anionic, respectively.

This selection enabled the investigation of the three extraction mechanisms and extraction priorities, as well as a better understanding of solution behavior with respect to the extractant class.

The experiments were carried out using the extractants at a concentration of 10% v/v diluted in kerosene. The aqueous-to-organic phase ratio was initially maintained at 1:1, and the contact time was 15 min under constant stirring at 25 °C, as shown in Table 1.

**Table 1:** Experimental Conditions and Parameters Evaluated in the Solvent Extraction Experiments after Citric Acid Complexation

extractant	pH	organic-to-aqueous phase ratio (O/A)	contact time (min)	temperature (°C)
D2EHPA	1	1/1	15	25
Cyanex 923	2			
Aliquat 336	3			

After the first sest of experiments aimed at studying complexation, the proportions of the added complexing agent were varied, Fe:cit equals 1:1 and 1:0.8. The same experimental procedure described above was performed in order to investigate the effect of varying the amount of complexing agent.

The extraction percentage was calculated according to eq 1.

1 $$\%=\frac{\left(C_{i}V_{i}\right)-\left(C_{f}V_{f}\right)}{\left(C_{i}V_{i}\right)}\cdot 100=\frac{\left(m_{i}-m_{f}\right)}{m_{i}}\cdot 100$$

where/*C*_i_ and *C*_f_ = metal concentration in the feed liquor and in the raffinate (mg/L), respectively; *V*_i_ and *V*_f_ = volume of the feed liquor and the raffinate (L), respectively; *m*_i_ and *m*_f_ = mass of the metal in the feed liquor and in the raffinate (mg), respectively.

The distribution coefficient (*D*) and separation factor (i) were also calculated according to eqs 2 and 3.

2 $$D=\frac{{\left[M\right]}_{org}}{{\left[M\right]}_{aq}}$$

The distribution coefficient relates the concentrations of the same metal in the organic ([M]_org_) and aqueous ([M]_aq_) phases.

3 $$S=\frac{D_{A}}{D_{B}}$$

The separation factor is the ratio of the distribution coefficients of metals A and B.

## Results and Discussion

### Elementary Characterization

Upon receipt, the solution was characterized to identify the elements present. The chemical characterization of the liquid fraction of the leach liquor was performed using ICP-OES and EDXRF, and the results are presented in Table 2.

**Table 2:** Elemental Composition of the Leach Liquor

Element	Concentration (mg/L)
Fe	25000
Total rare earth elements (Ln + Y)	2500
Y	750
La	29.55
Ce	185.46
Pr	7.73
Nd	83.19
Sm	70.68
Eu	2
Gd	117.22
Tb	62.44
Dy	460.41
Ho	48.19
Er	271.46
Tm	43
Yb	320.56
Lu	5.86

The concentration of rare earth elements in the leachate was approximately 2.5 g/L. The characterization indicated that Fe was the main contaminant in the studied solution, present at a concentration of 25 g/L. Other elements, including Nb, Ta, U, and Th, were also identified during the characterization of the leach liquor. However, these elements were not included in the proposed separation approach and, therefore, were not investigated in subsequent experiments. The scope of this study was specifically focused on evaluating the selective separation of rare earth elements from Fe, which represents the primary impurity in the investigated hydrometallurgical liquor.

The concentration of sulfate ions (SO_4_^2−^) was higher than that of all other elements present in the liquor, 337 g/L, and it comes from the hydrometallurgical process carried out using sulfuric acid. The overall Fe:Σ­(Ln + Y) ≈ 10:1 (mass basis) indicates a highly competitive medium.

The initial redox potential of the liquor was approximately 440 mV (0.44 V), and the pH was negative close to zero (−0.5).

### Redox Variation

The FactSage software was used to simulate the sample and determine the predominant species in solution as a function of pH and ORP. The resulting diagrams are presented in Figures 1 and 2. La was used to represent all elements of the group in Figure 2. As previously mentioned, the initial redox potential of the liquor was around 0.44 V, and the pH was near zero. According to the obtained diagram, the predominant Fe species in solution under these conditions was Fe^2+^.

**Figure 1:**
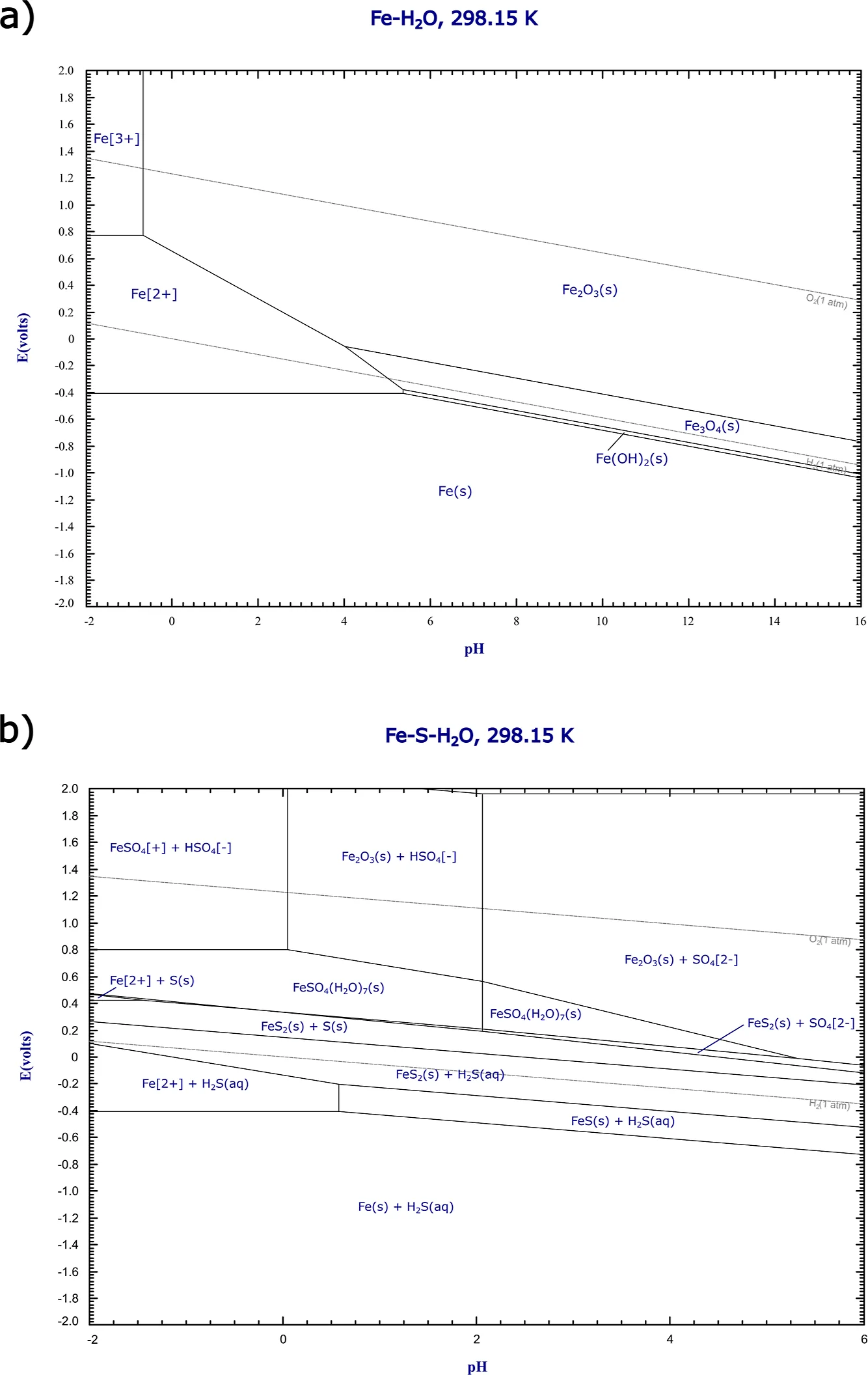
Fe predominance diagrams for (a) a system containing only water and (b) a sulfuric acid system.

**Figure 2:**
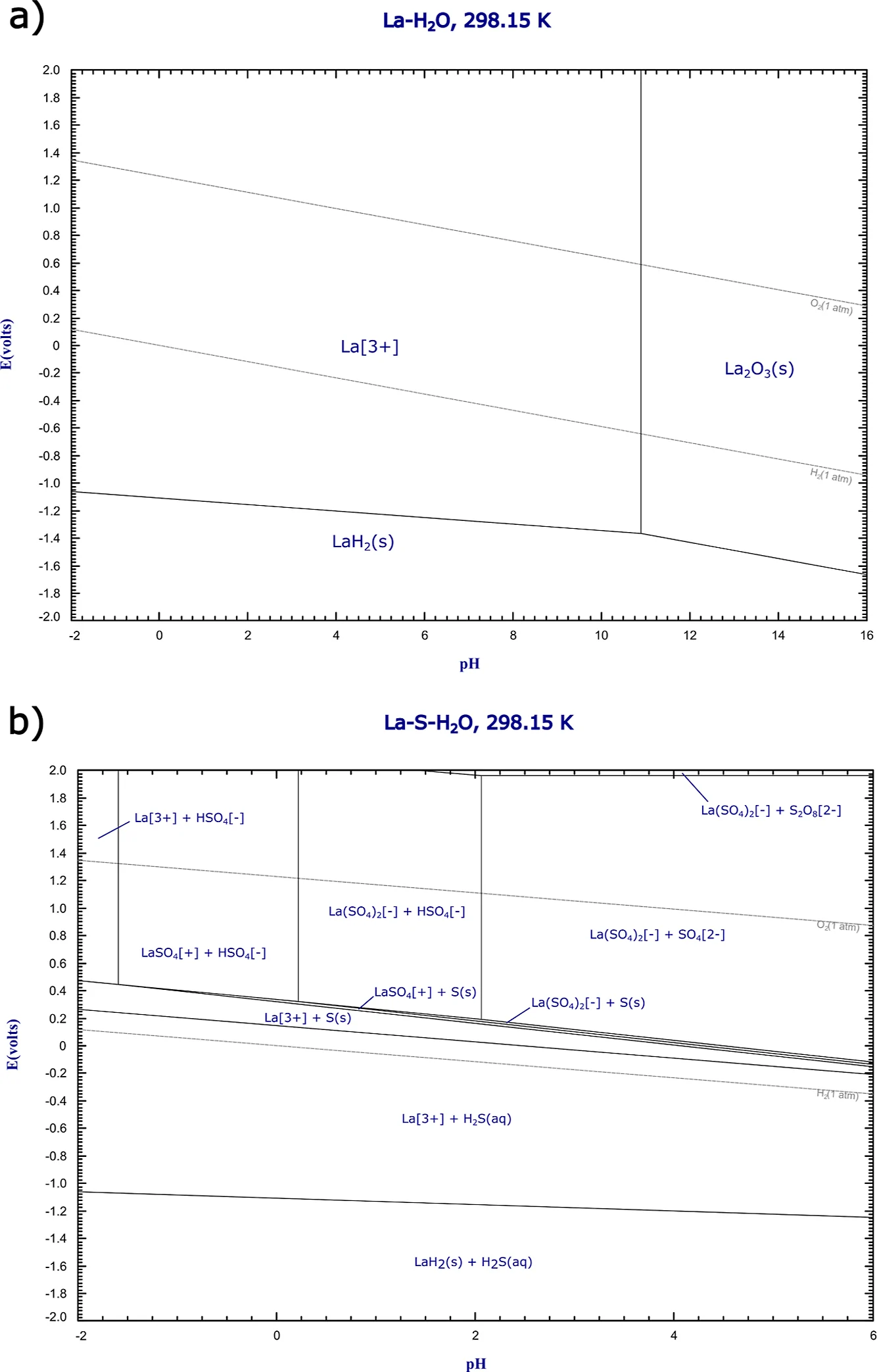
La predominance diagrams for (a) a system containing only water and (b) a sulfuric acid system.

According to literature^43^ it can be concluded that Fe precipitation in its divalent form occurs only at pH values above 8. The reduction of the redox potential was carried out to ensure that all Fe was maintained as Fe^2+^, and that selective precipitation of rare earth elements could be achieved. According to Figure 2, selective precipitation would be effective mainly because the reducing system within the studied pH range does not interfere with lanthanide species, since the only elements of this group that undergo reduction are Sm, Eu, and Yb. This allows precipitation in the form of rare earth-sodium double sulfates, for example.

The results from these experiments are shown in Table 3.

**Table 3:** Percentage of Rare Earth Elements and Fe Remaining in the Liquor after Redox Potential Adjustment with Na_2_S_2_O_4_ and pH Increase (1, 2, and 4.5)

	Percentage in solution (%)		
	pH = 1	pH = 2	pH = 4,5
Rare earth elements	88	83	82
Fe	100	90	71

However, when comparing the precipitation behavior in experiments conducted with and without the addition of the reducing agent, it was observed that the presence of Na_2_S_2_O_4_ in the solution influenced the formation of precipitates of both rare earth elements and Fe. The percentage of metals remaining in solution was higher when the liquor’s ORP was reduced. This result supports the adopted strategy, given the behavior of Fe, which did not precipitate before its theoretical precipitation pH (>8).

The experimental results indicated that simply maintaining a low redox potential and keeping Fe mostly in the divalent form were not enough to effectively precipitate rare earth elements selectively. Although Fe stayed mostly soluble at lower pH levels, raising the pH led to gradual Fe precipitation, and some rare earth elements were also removed from the aqueous phase. At pH levels 1–4.5, the proportion of Fe in solution dropped from 100 to 71%, while the rare earth fraction slightly decreased from 88 to 82%.

The incomplete precipitation of lanthanides can be explained by their oxidation states and the sulfur species present under the pH and Eh conditions studied. As indicated in eq 4, the precipitation of rare earth-sodium double sulfate requires the availability of Na^+^, which was supplied in excess in this study by the NaOH reagent, as well as SO_4_^2−^ ions in solution, in addition to the rare earth elements. Upon addition of the reducing agent to the solution, free SO_4_^2−^ ions may be reduced to sulfite (SO_3_^2−^), thiosulfate (S_2_O_3_^2−^), elemental sulfur (S^0^), or sulfide species (H_2_S/S^2−^).^44,45^ Species such as SO_3_^2−^ and S_2_O_3_^2−^ may not form the same desired double salt.

4 $$M^{+}+{RE}^{3+}+{2\mathrm{SO}}_{4}^{2-}+{xH}_{2}O↔MRE{\left({\mathrm{SO}}_{4}\right)}_{2}\cdot {xH}_{2}O$$

As can be observed in the diagram presented in Figure 2a, at Eh values of 0–0.2 V and pH values between 1 and 4, the predominant species are LaSO_4_^+^, La^3+^, and S(s), with the occurrence of La­(SO_4_)_2_^−^ within a narrow region of the diagram.

The Eh and pH range investigated in this study represents an unstable equilibrium region between the oxidized and reduced sulfur species.

The reducing environment contributed to incomplete or nondominant precipitation of rare earth elements within the pH range of 1–4.5, as reported in the literature, mainly due to the oxidation state of sulfur in the solution and the limited availability of SO_4_^2−^ ions. Considering only the behavior indicated by the simulated diagram, it can be inferred that further increasing the solution pH would likely result in a higher percentage of precipitation of these elements.

Selective precipitation of rare earth elements in a reducing system has been reported in the literature for Sm, Eu, and Yb, as these are the only elements that can exist in the divalent form in aqueous solution, enabling their separation from the others.^3,24,46^ However, in the present study, this behavior could not be observed due to the redox potential range investigated. The redox potentials of the Sm^3+^/Sm^2+^, Eu^3+^/Eu^2+^, and Yb^3+^/Yb^2+^ couples are approximately −1.55 V, −0.35 V, and −1.05 V, respectively; therefore, from a theoretical standpoint, these metals were not reduced under the experimental conditions.

The approach of varying the solution’s redox potential can also be used to separate Ce from other rare earth elements by adding an oxidizing agent to the solution to increase its oxidation state to the tetravalent form (Ce^4+^), a property unique to these elements within the group.^47,48^

### Complexation

The complexation study aimed to maintain the main impurity of the liquor, Fe, in solution so that the separation of rare earth elements could be achieved. Speciation diagrams of citric acid in the presence of sulfuric acid, which constitutes the sample matrix, were simulated using the Hydra/Medusa software and are presented in Figure 3.

**Figure 3:**
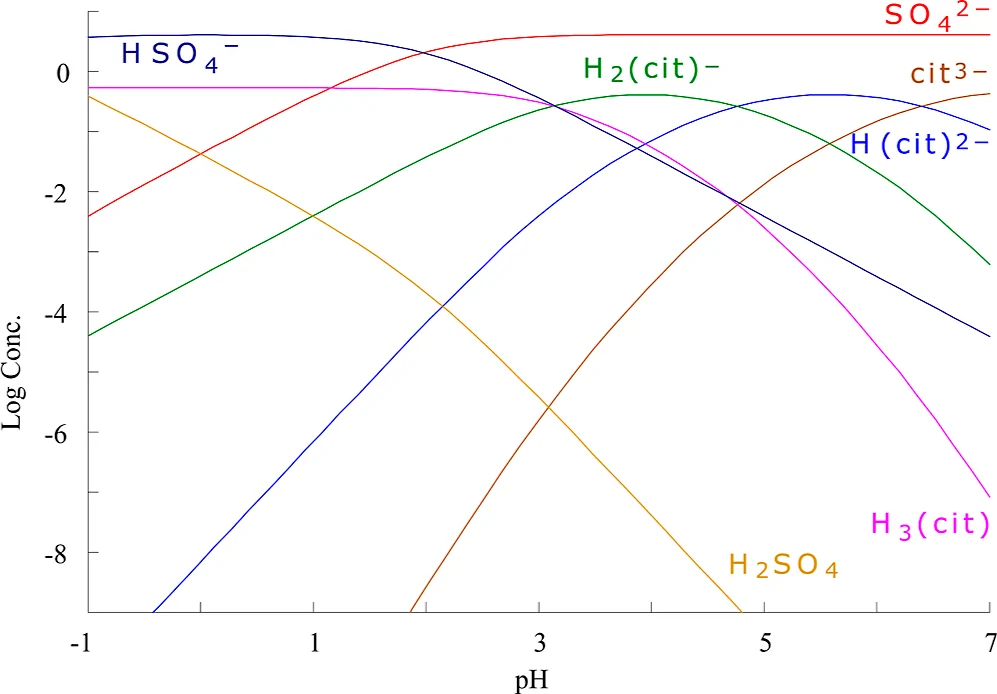
Citric acid speciation diagram as a function of pH in the presence of sulfuric acid.

These diagrams helped clarify how the complexing agents behave in the studied system and enabled the identification of potential species formed at different pH levels.

As shown in Figure 3, at low pH values, three citric acid species coexist at different concentrations: the fully protonated from (H_3_(cit)) is present at the highest concentration, followed by the species resulting from the first dissociation step (H_2_(cit)^−^) and that from the second dissociation step (H­(cit)^2−^). As the solution’s pH increases, the concentration of the fully protonated form decreases, while the (H_2_(cit)^−^) and H­(cit)^2−^ species increase, reaching their maximum in the pH range of 3−6. Complete deprotonation of citric acid (cit^3−^) begins to occur at approximately pH 2 and reaches its maximum above pH 6. Theoretically, the fully dissociated form is the most favorable for meal complex formation. The ionization reactions of the reagent are described in eqs 5–7.^18^

5 $$H_{3}cit↔H^{+}+H_{2}{cit}^{-}$$

6 $$H_{2}{cit}^{-}↔H^{+}+H{cit}^{2-}$$

7 $$H{cit}^{2-}↔H^{+}+{cit}^{3-}$$

The main contaminant in the solution investigated in this study, Fe, as well as La, used here to represent the lanthanides, was introduced into the Hydra/Medusa simulation to identify the possible species formed as a function of pH.

The diagrams were obtained by considering a system containing only the complexing agent and the metal discussed individually to facilitate visualization and interpretation, since including additional components in the simulation results in a greater number of curves and species in the diagram.

From the diagram in presented in Figure 4, it can be observed that the fully protonated form of citric acid and Fe^2+^ ion decrease with increasing pH, while complexed species such as Fe­(H_2_cit)^+^, Fe­(Hcit), H_2_(cit)^−^, and Fe­(cit)^−^ become more prevalent in solution within the studied pH range (1−3). For the system containing La and citric acid, it is observed that the only ion exhibiting a decrease in concentration within this pH range is La^3+^, whereas dissociated citric acid species, such as H_2_(cit)^−^ and H­(cit)^2−^, as well as the La­(cit) complex, become more abundant in the medium. The Hydra/Medusa simulation for this system does not indicate additional interactions between La and citric acid beyond the La­(cit) complex.^18,19,32^

**Figure 4:**
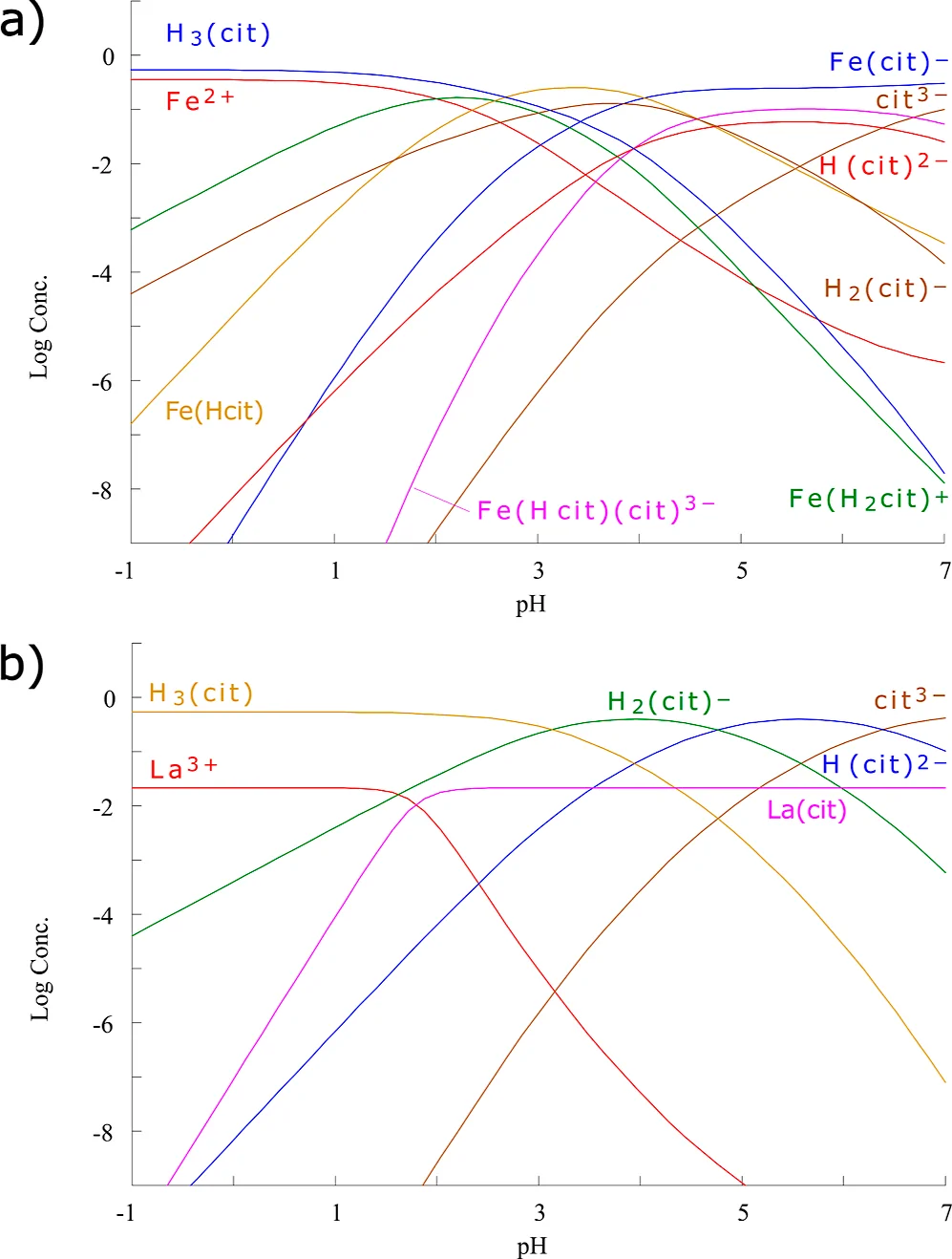
Speciation diagram of (a) Fe and (b) La species in citrate medium as a function of pH.

### Complexation Followed by pH Increase

According to Clarizia et al.^41^ Fe complexes with ligands such as citric acid reach maximum formation at pH values above 2. However, this pH value could not be attained before the addition of the complexing agent, since the predominant precipitation of rare earth elements and impurities occurs at pH values as low as 1. Therefore, the adopted approach consisted of adding the complexing agents to the solution, followed by increasing the pH with NaOH, thereby promoting complex formation and the simultaneous precipitation of rare earth elements, if they were not similarly complexed.

The percentage of metals remaining in the solution after the addition of the complexing agent and the subsequent increase in pH is presented in Figure 5. The presence of the ligand influenced the behavior of the ions investigated in this study.^49^ Even if stable complexes were not fully formed with the rare earth elements, since a fraction of these metals precipitated, the introduction of new species that increase the solubility of metallic ions affected their behavior. This influence is particularly evident for Fe^2+^, which was completely maintained in solution when complexed with citric acid.

**Figure 5:**
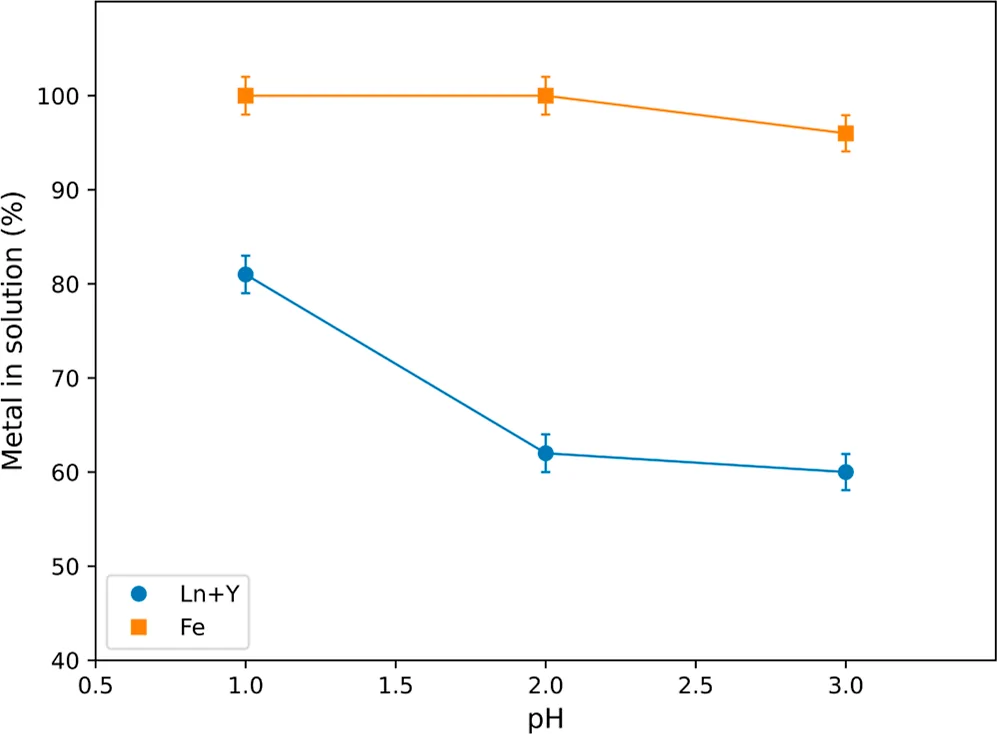
Percentage of rare earth elements and Fe maintained in solution after citric acid addition and pH adjustment.

In the experiment conducted with citric acid in excess, its presence reduced the percentage of rare earth precipitation compared with experiments performed in its absence (previously conducted by the group).^32^ Nevertheless, precipitation increased with increasing pH. At pH 1, citric acid is fully protonated, as indicated in Figure 4, and does not directly interfere with the precipitation of rare earth double sulfates; therefore, these metals are partially precipitated (approximately 20%), but also remain in solution as the soluble complex (Ln + Y)­SO_4_^+^. As the pH increases, citric acid undergoes deprotonation, and lanthanide complexation begins, which would interfere with precipitate formation. At pH 3, citric acid partially loses protons, forming citrate ions with negatively charged carboxylate groups that can bind metal ions in water. The complexation of rare earths occurs through coordination between the (Ln + Y)^3+^ ions and the oxygen donor atoms of the deprotonated carboxylate groups, forming soluble (Ln + Y)­(cit) complexes. This interaction reduces the activity of free (Ln + Y)^3+^ ions in solution and favors the formation of complexed forms. The degree of complex formation depends on citric acid’s acid−base equilibria and the stability constants of the related metal-citrate complexes.

Organic acids exhibit reducing behavior in solution; thus, under the conditions of pH 2 and Eh 0.33 V, and at pH 3 and Eh 0.15 V, rare earth elements are predominantly present as (Ln + Y)­(SO_4_)_2_^−^, considering the liquor system, and SO_4_^2−^ ions remain available in the medium, leading to an increase in the percentage of precipitation.^50^

The direct interference of cit^3−^ species with rare earth elements is further reduced by the presence of Fe in solution. Although the formation constant of the complex between rare earth elements and citric acid (log *K* = 7.1–8.07) is slightly higher than that of the Fe–citrate complex (log *K* = 4.85), the higher concentration of Fe means that the concentration parameter exerts a greater influence, leading predominantly to the formation of Fe-citrate complexes. Given the complexity of the multielement system, it can be concluded that multiple species coexist in the medium, with simultaneous competition between free and complexed forms under equilibrium conditions.^51^

### Solvent Extraction after the Addition of a Complexing Agent

After the addition of the complexing agent in stoichiometric excess relative to Fe and the subsequent increase in solution pH, the solutions were filtered and subjected to solvent extraction experiments using D2EHPA, Cyanex 923, and Aliquat 336. The extraction percentages obtained in these experiments are presented in Figure 6.

**Figure 6:**
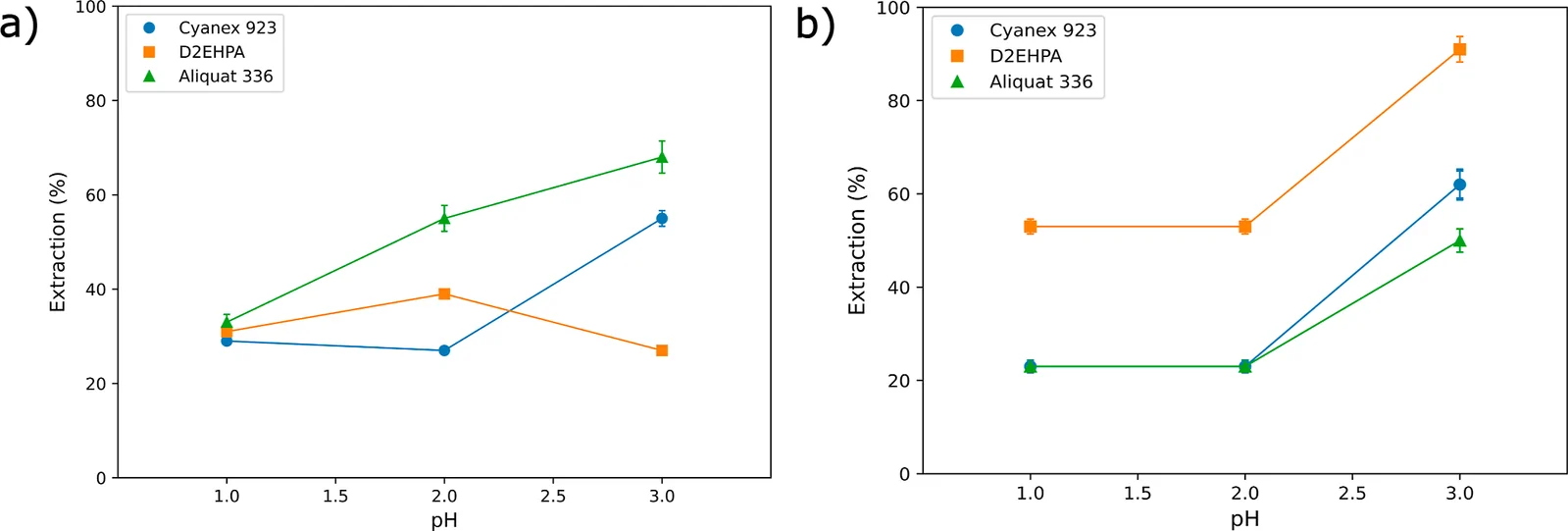
Extraction of (a) Fe and (b) rare earth elements using Cyanex 923, D2EHPA, and Aliquat 336 at pH 1, 2, and 3.

Solvent extraction in citric media or in systems containing citrate species has been reported in the literature for both the separation of metals in solution and the selective extraction of citric acid itself.^52–56^

The behavior of the three evaluated organic solvents indicates higher extraction percentages as the solution pH increases, for both rare earth elements and Fe. At pH 1, Fe extraction was favored, possibly due to its higher concentration relative to rare earth elements and the minimal presence of neutral complexes. When the solution reached pH 2, no significant change in the extraction profile of the studied metals was observed. However, a marked increase in extraction percentage was noted at pH 3 compared with lower pH values. In all experiments, extraction values were similar at pH 1 and 2; nevertheless, as the aqueous phase pH approached 3, an increase of up to 40 percentage points in rare earth extraction was observed.

The extraction mechanism of Cyanex 923 is based on solvation, that is, the transfer of neutral species from the aqueous phase to the organic phase occurs through solvation of the proton of an acidic metal complex species or of a metal ion from a neutral salt.^57^ Therefore, based on the results obtained with this extractant, the highest Fe extraction at pH 3 can be mainly attributed to the presence of neutral Fe-citric acid complex species in solution (Fe­(Hcit)). The possible existence of Fe­(cit)^−^ complexes cannot be excluded; however, their formation constant is relatively low (4.8) compared with the stability of the species formed with Fe^3+^ (11.5), which favors the release of Fe, allowing it to be complexed and subsequently extracted by the extractant.^58,59^

It is also important to emphasize that the disproportionately high concentration of Fe in the solution relative to the lanthanides likely compromises the selectivity reported in the literature and may lead to partial saturation of the organic phase. Under such conditions, competitive extraction effects become significant and can limit the effective transfer of rare earth elements to the organic phase. The higher rare earth extraction observed at this pH suggests the presence of neutral species such as (Ln + Y)­(cit) or sulfate-containing complexes; however, it is unlikely that these elements are predominantly present in fully neutral forms, as might be anticipated based on the previous precipitation step. If neutral (Ln + Y)­(cit) species were the dominant forms in solution, Cyanex 923 would be expected to exhibit markedly higher extraction efficiency than the other extractants evaluated. The absence of such behavior indicates that speciation and intermetallic competition in the multicomponent system play a decisive role in controlling the extraction process.

Aliquat 336 exhibited an extraction behavior opposite to that of Cyanex 923 and D2EHPA, preferentially extracting Fe over rare earth elements. This result suggests the formation of anionic species between Fe and the complexing agent, such as Fe­(Hcit)­(cit)^3−^ and Fe­(cit)^−^, or between Fe and components of the medium, corroborating the behavior previously reported by Araújo et al. (2017).^52^ Because the extraction percentages obtained in experiments using Aliquat 336 were higher for Fe than for rare earths, separation factors were also calculated using the Fe-to-rare earth ratio (S_Fe/Ln+Y_). For experiments conducted at pH 1, 2, and 3 using Aliquat 336, the S_Fe/Ln+Y_ values were 1.61, 4.16, and 2.16, respectively. These results indicate that although pH 3 enabled higher Fe extraction, the greatest separation between Fe and the lanthanide group was achieved at pH 2.

It can be concluded that, under the same experimental conditions, the effectiveness of the extractants is strongly influenced by the extraction mechanism involved and by the predominant species in the solution. D2EHPA exhibited the highest selectivity toward rare earth elements, emerging as the most promising extractant for this system. In contrast, Cyanex 923 promoted coextraction of Fe, whereas Aliquat 336 predominantly extracted Fe due to the prevalence of anionic species within this pH range.

### Effect of Complexing Agent Dosage on Metal Precipitation

After conducting experiments under excess complexing agent conditions, the dosage was systematically varied to assess the influence of the ligands in solution.

Citric acid was added based on the Fe concentration in the sample, using stoichiometric (Fe:cit 1:1) and substoichiometric (Fe:cit 1:0.8) ratios. The results of the first stage (pH adjustment) are presented in Figure 7.

**Figure 7:**
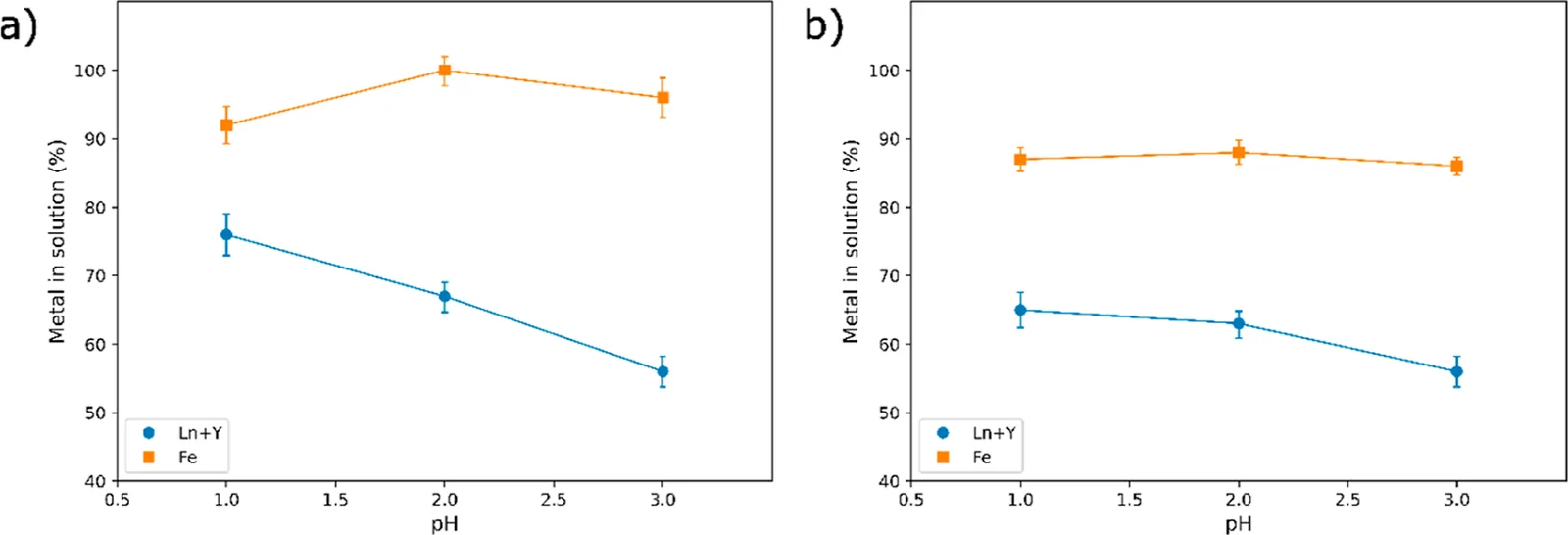
Percentage of rare earth elements and Fe remaining in solution after citric acid addition at different ratios, (a) Fe:cit 1:1 and (b) Fe:cit 1:0.8, followed by pH adjustment.

Compared with the experiment performed with an excess of complexing agent, it is observed that citric acid produced a weaker effect on the system, as the precipitation of rare earth elements and Fe was higher in the experiments with lower ligand concentrations.^32^

In the experiment conducted at pH 1, the percentage of rare earth elements remaining in the liquor increased with increasing reagent addition (1:0.8 = 65% < 1:1 = 76% < 1:1.5 = 81%). A similar trend was observed for Fe, with 87% remaining in solution when citric acid was added in a substoichiometric proportion and reaching 100% when the complexing agent was in excess in the solution.

At pH 3, the highest precipitation percentages of the evaluated elements were observed compared with the other pH values, as previously discussed. It can therefore be concluded that, although the pH reached is theoretically responsible for promoting lanthanide precipitation, the added ligand tends to undergo deprotonation, thereby increasing the likelihood of forming soluble complexes.

The experimental results once again highlight the influence of the diversity of metals present in the sample and the concentration of impurities, which affect both the selectivity of complex formation and the tendency to precipitate.

### Effect of Complexing Agent Dosage on Solvent Extraction

The second stage of the study (solvent extraction tests) was conducted exclusively with D2EHPA because the highest extraction observed in the experiments discussed in the previous section was achieved with the cationic extractant. The results obtained for Fe:cit ratios of 1:1 and 1:0.8 are presented in Figure 8.

**Figure 8:**
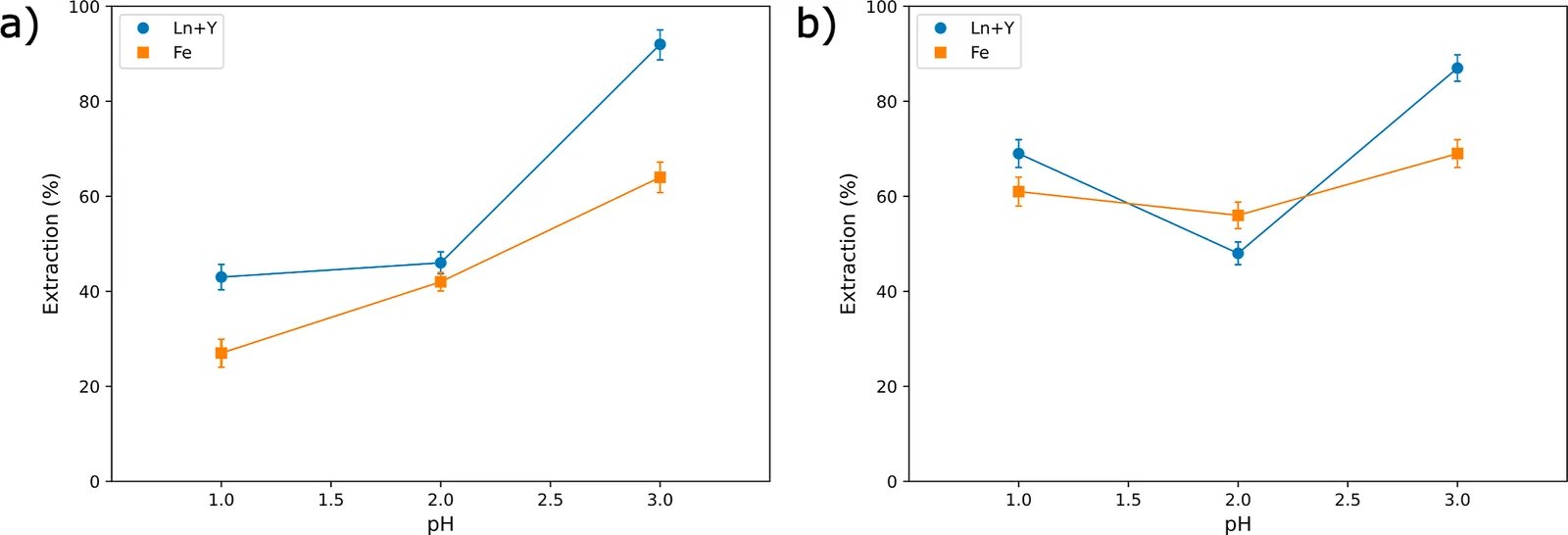
Extraction of rare earth elements and Fe using D2EHPA after citric acid addition at different ratios, (a) Fe:cit 1:1 and (b) Fe:cit 1:0.8, and pH adjustment.

According to the results presented in Figure 8, there is a sharp increase in the extraction percentage of rare earth elements as the solution’s pH increases, similar to the experiment conducted with excess citric acid. Although the extraction of these metals (92%) is comparable to that under stoichiometric excess conditions (91%), the extraction of Fe is higher (64%) than in the previous experiment (27%). The similar extraction levels of lanthanides in both cases suggest a preferential interaction of the ligand with the contaminants rather than with the rare earth elements. This also indicates competition among impurities in the liquor for the ligand; consequently, the excess complexing reagent added provides enough deprotonated ligand species, preventing subsequent extraction by the cationic extractant.^60^

This hypothesis is further supported by experiments using substoichiometric amounts of the ligand, in which Fe extraction was higher than in tests with higher citric acid concentrations, especially at pH 3 (69%). In contrast, the extraction of rare earth elements decreased only slightly, by approximately five percentage points (87%).

The complexation study, serving as a supplementary method to another purification process, demonstrated effectiveness in isolating rare earth elements from Fe in the leachate, especially when contrasted with experiments that did not include a complexing agent.^60–63^ This is supported by the high rare earth extraction percentage (91%) and notably by the separation factor (27) achieved in the experiment with excess citric acid addition (Fe:cit 1:1.5) at pH 3, followed by solvent extraction with 10% v/v D2EHPA. Consequently, these conditions were deemed the most advantageous in this study.

### Rare Earth Elements and Fe Distribution along the Separation Route

The initial sample had a rare earth element-to-Fe concentration ratio ((Ln + Y)/Fe) of 0.1, indicating that the original leachate had a high Fe concentration relative to the concentration of rare earth elements.

Following the initial stage of the proposed separation route—adding excess citric acid and adjusting the pH to 3—a solid phase was produced with an (Ln + Y)/Fe ratio ten times higher than the original. This indicates the success of the selective precipitation step in lowering Fe contamination and enriching the rare earth elements in the precipitate.

In contrast, the residual liquor after this stage had an approximate (Ln + Y)/Fe ratio of 0.0625, suggesting that a substantial part of Fe stayed in solution through complexation with citric acid.

The subsequent solvent extraction stage, carried out using the filtered liquor and 10% v/v D2EHPA, produced two distinct streams: an aqueous raffinate with the lowest lanthanide concentration across all stages of the process and an organic phase with an (Ln + Y)/Fe ratio approximately twice that of the initial leachate. Although Fe remained present in the loaded organic phase, the higher proportion of rare earth elements relative to the contaminant indicates that the extraction system promoted selective enrichment of rare earths.

These results highlight the synergistic effect between aqueous complexation and solvent extraction, in which citric acid selectively suppresses Fe extraction while maintaining high transfer of rare earth elements to the organic phase. The integrated approach therefore improved the selectivity of the separation process and contributed to generating a more concentrated rare earth stream from the columbite leachate.^32,60,63,64^

Based on the results obtained, another possible alternative is a route in which the pH is adjusted to 2 after the addition of an excess of the ligand. Under these conditions, 100% of the Fe remained in solution, while approximately 40% of the rare earth elements precipitated. The production of this Fe-free solid may therefore represent a second viable approach for separating the elements.

## Conclusion

The results showed that the reduction of the solution potential, carried out to maintain Fe in its divalent form, did not contribute to the selective precipitation of rare earth elements. The availability of SO_4_^2−^ species was altered, preventing the formation of Na­(Ln + Y)­(SO_4_)_2_.

The addition of a complexing agent to the leachate promoted the formation of species that significantly influenced the precipitation behavior and liquid−liquid extraction of the studied metals. The use of excess citric acid (Fe:cit = 1:1.5) kept 100% of Fe in solution across the pH range of 1−2, while simultaneously promoting the precipitation of a fraction of the rare earth elements (20 and 40%, respectively). As a result, an Fe-free solid phase was obtained.

The evaluation of citric acid concentrations showed that an excess (Fe:cit = 1:1.5) is required to effectively mask Fe during extraction with D2EHPA. At pH 3, the Fe extraction percentage decreased from 69 to 27% as the Fe:cit stoichiometric ratio increased from 1:0.8 to 1:1.5. Under the same conditions, 91% of the rare earth elements were extracted, yielding the highest separation factor reported in this work (26), compared with previous experiments conducted by the research group without the addition of complexing agents. These findings highlight the effectiveness of complexation as an auxiliary strategy in separation and purification processes.
